# A hybrid approach to large-scale systematic literature reviews: combining automated tools with text-mining techniques

**DOI:** 10.1186/s13104-026-07651-7

**Published:** 2026-01-30

**Authors:** Zhao Hui Koh, Armita Zarnegar, Jason Skues, Greg Murray

**Affiliations:** 1 Centre for Mental Health and Brain Sciences, Swinburne University of Technology, Hawthorn campus, John Street, Hawthorn, VIC 3122 Australia; 2 Department of Psychological Sciences, Swinburne University of Technology, Hawthorn campus, John Street, Hawthorn, VIC 3122 Australia; 3 School of Software and Electrical Engineering, Swinburne University of Technology, Hawthorn campus, John Street, Hawthorn, VIC 3122 Australia

**Keywords:** Preliminary screening, Systematic review, Automated tools, Text-mining, Machine learning, Natural language processing, Expert knowledge, Automation, Large-scale reviews

## Abstract

**Objective:**

Semi-automated tools used during the preliminary screening of articles in systematic reviews can start with a small set of seed articles and actively learn from human decisions to prioritise more relevant articles for subsequent screening. However, given that these tools are vulnerable to biases and lack clear stopping criteria, their performance in large-scale systematic reviews remains uncertain, especially in reviews covering broad subject areas that require a substantial number of representative seed articles. This article presents a hybrid approach that uses text-mining techniques combined with a semi-automated tool to effectively reduce, screen, and validate a large cohort of articles (*N* = 90,871).

**Result:**

A preliminary evaluation using simulations indicated that this approach has the potential to craft a comprehensive collection of seed articles that covers broad subject areas for semi-automated tools in a large-scale systematic review. The strengths and limitations of using a semi-automated tool alone in such a context are discussed. Our approach increases the efficiency of automated tools by providing a larger and more focused selection of articles to start with, optimising the learning process for those tools and reducing biases. Additionally, our approach could increase the transparency and reusability of keywords for future review updates.

**Supplementary Information:**

The online version contains supplementary material available at 10.1186/s13104-026-07651-7.

## Introduction

Automation tools can reduce the screening effort in systematic reviews, a task previously deemed time-consuming [1–3], by 30%−50% with only a marginal precision loss (up to 5%) [4–6]. This promising performance could address concerns regarding the quality and reproducibility of research evidence [7, 8] while meeting the demand for faster and more extensive reviews. However, a fully automated tool must train with many articles and associated human decisions to reach an acceptable level of accuracy [9, 10]. This is impossible for a *new* systematic review with few screened articles.

Semi-automated tools (e.g., ASReview [11], Rayyan [12]) can start with limited seed articles, learn the decisions in vivo during screening and re-prioritise the relevance of the remaining articles [13]. Although their prospect seems promising [14], they are susceptible to the *hasty generalisation problem*, a bias in which a tool overgeneralises articles based on the information it has learned so far [4, 6, 15]. Furthermore, semi-automated tools typically require a stopping criterion for screening to be defined (e.g., the number of consecutive irrelevant articles screened) [16], which requires expert judgement. Stopping prematurely during screening could lead to missing a substantial number of eligible articles, which is a key drawback of relying solely on this approach [9]. Amidst these limitations, it is unclear how effective these tools are in large-scale reviews investigating broad subject areas, especially when a representative set of starting articles covering areas of interest is desirable.

This article outlines a hybrid approach combining text-mining and a semi-automated tool to undertake preliminary screening within a large-scale systematic review with broad subject areas (in press; see the protocol [17] for more details; PROSPERO ID: CRD42022306547). Our review [17] was designed to investigate empirical studies that administered mental health instruments in (1) the general population, (2) digital format, and (3) longitudinal designs. The database searches yielded approximately 95,000 unique articles, which were infeasible to screen serially. An early investigation using semi-automated tools to facilitate screening was unsatisfactory due to concerns about missing articles given the broad review.

## Methods

In a systematic review focused on a broad topic, search results often return many irrelevant articles, making manual screening challenging. Using broad search terms in the database query resulted in numerous articles that included the target terms but were unrelated to the topic of interest (see Appendix 1 for some examples). Since it was impractical to screen all articles *serially* and include all permutations of *negative keywords* in the search query, we developed a method using text-mining techniques, such as keyword matching [18, 19], combined with domain knowledge and expert-weighted criteria to identify relevant articles (described below). We used this approach to screen *all* articles in our review.

In this section, Part 1 describes our approach in detail and Part 2 explains how the approach was provisionally evaluated. Based on the evaluation results, we highlighted the potential utility of our approach for identifying representative articles to use as input (seed) for semi-automated screening tools, thereby enhancing the tools’ capabilities.

### Part 1: the term-scoring approach

**Fig. 1:**
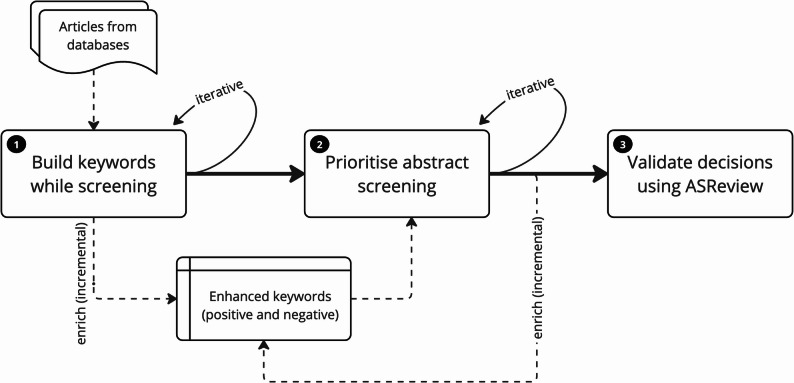
Approach in preliminary screening titles and abstracts. The screening process involved (1) building up a list of positive and negative keywords to eliminate irrelevant articles by their titles; (2) assigning weights iteratively to the positive and negative keywords and calculating term scores to prioritise abstract screening; and (3) comparing screening results with ASReview. Both Step 1 and Step 2 involved text-mining and manual review. Step 3 used ASReview.

Our approach was a two-step iterative method (see Fig. 1). In Step 1, we eliminated irrelevant articles by their titles using positive and negative keywords that we generated and refined iteratively. Step 1 (title-only screening) reduced *noise* from the additional text in the abstracts, enabling easy identification of irrelevant articles. In Step 2, we calculated a *term score* for each article using (i) the article’s title and abstract, (ii) keywords generated in Step 1 and (iii) weights assigned to the keywords (between 1 and 10) by an expert based on their assessment of Step 1 to reflect their importance. These weights were revised iteratively during the screening process. The *term scores* were then used to prioritise the order of the remaining articles for screening. We used a simple scoring formula to calculate the term score (*TS*):

$$\:TS=\sum\:_{p=1}^{P}{w}_{p}{m}_{p}-1.2\left(\sum\:_{n=1}^{N}{w}_{n}{m}_{n}\right)$$

where

$\:P$ is the count of positive keyword matches

$\:N$ is the count of negative keyword matches

$\:{w}_{p}$ is the $\:weighting/10$ for the positive keyword

$\:{w}_{n}$ is the $\:weighting/10$ for the negative keyword

$\:{m}_{p}$ is the product of the match count and the word count for the positive keyword, over the word count in the title and abstract

$\:{m}_{n}$ is the product of the match count and the word count for the negative keyword, over the word count in the title and abstract

The aforementioned approach combined semi-automated rapid screening with iterative scoring to improve accuracy and efficiency in article selection. Each iteration enriched the keyword list and weightings, which re-calculated the articles’ term scores and re-ranked them. The process continued until all articles were screened. Upon completion, we cross-validated the screening results with ASReview (version 1.0) [11]. The cross-validation used approximately 70% of all screened articles as seeds (input) for ASReview. Once ASReview processed (learnt from) these seed articles, the remaining 30% of the articles were ranked by ASReview. We expected that the ranking of these articles would be associated with human decisions using the term-scoring approach described above. Appendix 1 elaborates more on the cross-validation procedure.

## Part 2: evaluation of the term-scoring approach

To demonstrate the validity of our approach, after the screening was completed, we ranked all articles by their term scores (called *term score ranking*) before analysing them. To compare our approach with using ASReview alone, multiple configuration profiles were set up in ASReview for simulations by varying the following parameters:

1. *Starting seed:* the number of seed articles provided to train ASReview, operationalised as the number of relevant and irrelevant articles.

2. *Mode:* could be either *active-learning mode* or *rank-once mode*. In the *active-learning mode*, a person actively screens the articles while ASReview actively learns about their decisions. In the *rank-once mode*, ASReview ranks the rest of the *un-screened* articles once, based on the seed articles.

The four configuration profiles were:

1. *Config A: *This configuration emulated the active learning using ASReview with minimal seed (1 relevant and 1 irrelevant article). Once the seed was loaded, the reviewer screened approximately 20 articles using the ASReview user interface.

2.* Config B: *This configuration loaded 20 relevant and 20 irrelevant seed articles to ASReview before ASReview ranked the rest of the unscreened articles once.

3. *Config C:* This configuration loaded 50 relevant and 50 irrelevant seed articles to ASReview before ASReview ranked the rest of the unscreened articles once.

4. *Config D:* This configuration represented how we cross-validated our screening effort (see Appendix 1). We loaded approximately 5,332 relevant and 58,480 irrelevant seed articles to ASReview before ASReview ranked the rest of the unscreened articles (*n* = 27,119) once. After that, we compared the ASReview rankings of these unscreened articles with our screening decision.

Once the simulation for each configuration was completed, the article rankings from ASReview were extracted for analysis. Additional simulation analyses examining ASReview’s recall rates and stopping criteria are provided in Appendix 2.

## Results

### Part 1 - term score ranking between eligible and ineligible articles

**Fig. 2:**
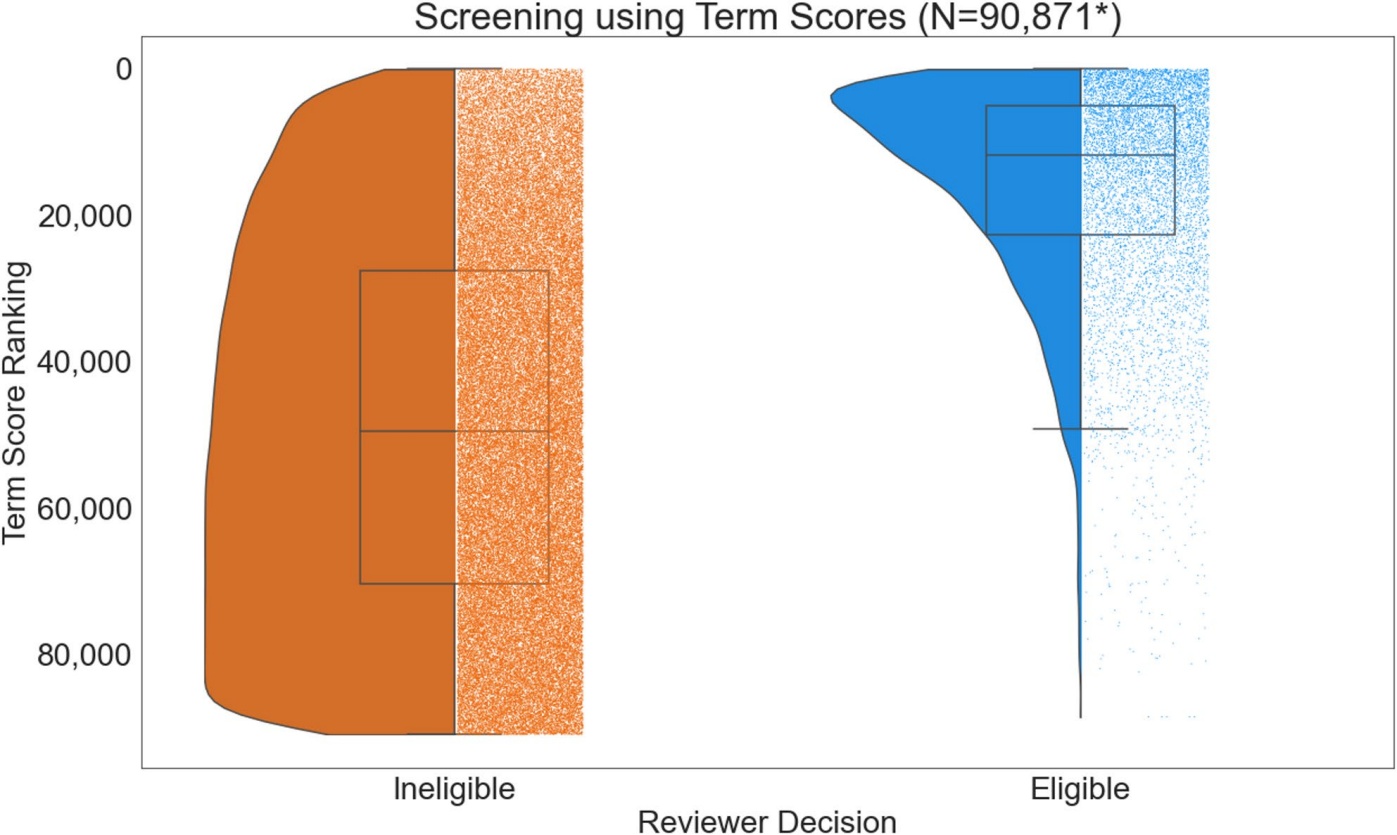
The distribution of term score ranking between eligible and ineligible articles. The y-axis is inverted. Lower-ranked articles are more relevant to the review. *Any articles with no DOI, no abstract, ineligible article types or abstracts with fewer than 200 characters (approximately 5% of the total number of articles) were considered ineligible and were ranked last with higher term-score ranking values.

To validate our term-scoring approach, first, we compared the ranking of term scores of articles grouped by their eligibility (see Fig. 2). In Fig. 2, the y-axis is inverted to represent ranking. A lower ranking implied a higher relevance of the article to the review. Our term-scoring approach yields a high percentage of true positives, with 75% of eligible articles appearing in the top quartile of ranked results. In contrast, ineligible articles showed a more dispersed ranking distribution. Manual inspection revealed that many ineligible articles contained a high number of positive terms but were excluded based on predefined criteria outlined in our systematic review (see [17]). Eligible articles that were missed in this screening stage will be picked up in full-text screening subsequently.

### Part 2 - ranking across different configurations

**Fig. 3:**
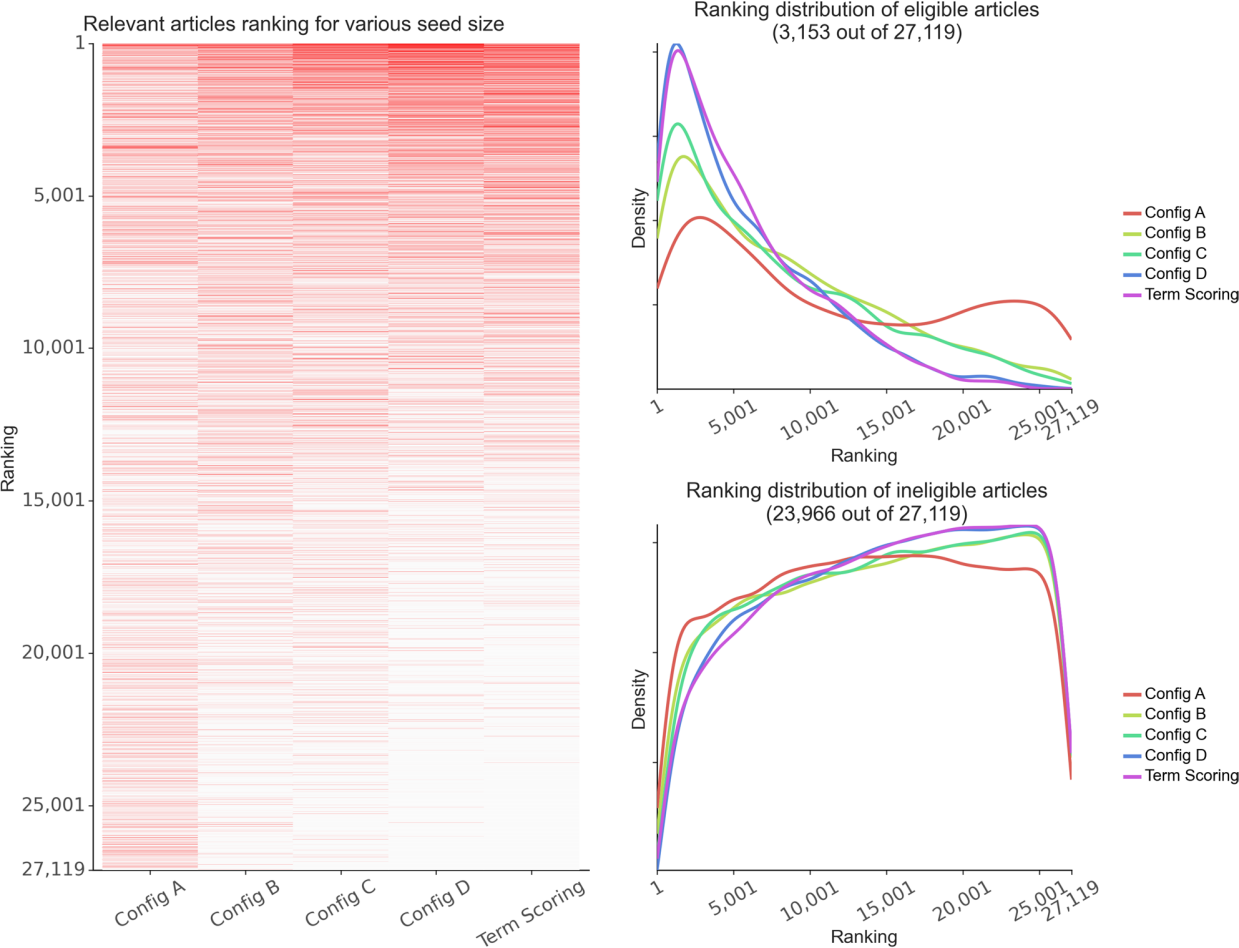
Ranking comparison across different configurations and term scoring. The red lines in the left subplot represent eligible articles. Config A: active learning mode with minimal seed (1 relevant and 1 irrelevant articles) and manual screening for 20 articles using ASReview; Config B: rank-once mode (i.e., ASReview ranked the *un-screened* articles *once* based on the seed articles) with 20 relevant and 20 irrelevant articles; Config C: rank-once mode with 50 relevant and 50 irrelevant articles; Config D: rank-once mode with 5,332 relevant and 58,480 irrelevant articles (Step 3 in Fig. 1); Term Scoring: term-scoring approach described above. The top-right subplot in Fig. 3 shows that Configuration D and the term-scoring approach yielded the narrowest ranking distributions, indicating more consistent performance compared to other configurations. The bottom-right graph reveals that ineligible articles were generally ranked lower under both Configuration D and the term-scoring approach. Notably, Config D and the term-scoring approach produced more accurate and concise results compared to other methods. In Configuration A, relevant articles (represented by red lines) were dispersed across the entire spectrum, suggesting less effective prioritisation. The ranking distributions between Configuration D and our term-scoring approach were highly similar, further validating the effectiveness of our term-scoring approach. In both approaches, the majority of eligible articles had lower rankings (indicating more relevance). The Spearman rank-order correlation [20] between Configuration D and the term-scoring approach was moderate ($\:\rho\:$ = 0.587, $\:p$ < 0.001), despite each using different mechanisms to screen the articles. Additional analyses of the ranked articles and a simulation-based evaluation of ASReview’s full-screening performance are detailed in Appendix 3 and Appendix 4 respectively.

To further validate the term-scoring approach, we cross-validated our screening effort by including 70% of randomly selected articles ($\:n$ = 63,752; 5,332 relevant, 58,420 irrelevant) as seed articles (as training data) in ASReview. The remaining 30% of articles ($\:n$ = 27,119) were used for testing. The results of this process are presented as Configuration D in Fig. 3. We compared this configuration to Configurations A, B and C, as well as to the term-score ranking alone (for details of each configuration, see Part 2 under Methods).

## Discussion

This article outlines a hybrid approach to preliminarily screen articles for a large-scale systematic review with broad subject areas and the provisional evaluation of this approach. One key distinction between our approach and the use of semi-automated tools alone is that our approach enables domain experts to directly influence the keywords and weightings, resulting in more accurate information early on (i.e., relevant seed articles) for subsequent prioritisation and, therefore, more efficient screening. Furthermore, by having a more representative set of seed articles for semi-automated tools, it has broader coverage and could prevent eligible articles being missed from premature stopping. In addition, should a decision is made to stop screening using a semi-automated tool, the keywords and term scores generated from this approach can be used to evaluate the relevancy of the unscreened articles. This is particularly crucial in a broad large-scale review. Our approach can be implemented as a standalone tool for abstract and title screening, or as a pre-screening tool to gauge the nature of the articles in broad-topic reviews and help tailor subsequent screening strategies, for example, integrating with a semi-automated tool (as demonstrated in this study).

For clarification, we do not claim that our approach is more efficient than using semi-automated tools in general. Rather, in a large-scale review covering broad subject areas, utilising the term-scoring approach to generate a set of relevant seed articles for semi-automated tools is more efficient than using a semi-automated tool alone. For instance, if the average number of titles and abstracts screenable daily is approximately 470 [21], then manually screening 60,000 seed articles in Configuration D would take approximately 128 days. The proposed hybrid approach can reduce the screening time.

The proposed approach has several limitations. Firstly, steps outlined here, such as weighted keywords, are domain-specific, so for each review an expert in the field needs to reassign the weights, which makes generalisability somewhat *limited*. Furthermore, in the present study, only one expert reviewer was involved in generating keywords and weightings, which could introduce biases during the screening process. Secondly, the iterative keyword refinement process in our approach is currently resource-intensive; a user-friendly interface should be developed to enable non-technical users, who may not be familiar with custom scripts, to perform it more easily. Future tool developers could consider providing a more generic approach that allows reviewers to identify domain-specific keywords and weightings to guide their review. Thirdly, although we screened all articles using the term-scoring approach, in hindsight, a potential improvement would be to incorporate the term-scoring approach at the start of the review for representative seed articles and integrate a semi-automated tool throughout the review process to guide the stopping criteria. Future studies could refine the hybrid approach introduced here, which harnesses the best of both worlds.

Amidst flourishing research in machine learning, it is not our intention in this article to devise a classification method to screen articles. We opted for a simple scoring mechanism that emerged from our real-world problem of screening articles for an otherwise overwhelming review task. We simply aim to demonstrate how text-mining techniques can be combined with automated tools to make screening decisions explicit (using keywords and weightings) for a large-scale review covering broad subject areas while improving both efficiency and transparency.

## Supplementary Information

Supplementary Material 1.

## Data Availability

The data and materials of this study are available from the corresponding author upon reasonable request.

## Appendix 1 method details of part 1

This section describes supplementary information on Part 1 of our screening process.

### Examples of irrelevant articles

Examples of irrelevant articles containing the target search terms unrelated to the constructs of interest in our review:

• “material **stress**”, “residual **stress** railroads”, “surface **stress**” (stress is one of the constructs of interest but not “material stress”; although we can restrict the search term to ‘psychological stress’, not all articles will use the term ’psychological stress’ consistently, for example, carer’s stress).

• “**wave**let”, “ocean **waves**”, “ultrasonic **wave**” (we are interested in studies that conducted mental health questionnaires in multiple *waves*).

• “forest **resilience**”, “infrastructure **resilience**”, “cyber **resilience**” (similar to the term *stress* above, we are interested in ‘psychological resilience’).

During Step 1 of the screening process (title only, see above), articles with titles matching the terms listed above (operationalised as negative keywords — regular expressions of the terms) were excluded. During Step 2 of the screening process (title and abstract, see above), the terms above were added to the negative keyword list to prioritise article screening if the terms appeared in many articles’ abstracts, for example, using the expression “wavelet[s]*” and “physiological stress” with a maximum weight of 10.

### Text-mining techniques and enhancements

During the article screening, two text-mining techniques: the *reviewer terms* and *automatic classification* [24] were employed. By definition, the *reviewer terms* technique involves reviewers defining relevant terms (positive keywords) and irrelevant terms (negative keywords). The *automatic classification* technique learns from a set of pre-screened articles based on some heuristic (e.g., the ratio between relevant and irrelevant terms for each article) before building a statistical model to infer the relevancy of unscreened articles.

We enhanced the *reviewer-terms* technique by allowing the keywords to be expressed as “regular expressions” [25] and assigning numerical weights (an integer between 1 and 10) to each keyword based on how important they are in determining the relevance of an article in this review. Regular expressions are a type of query syntax commonly used in computer programming to enhance text querying and matching. They are similar to the wildcard syntax used in the literature database search and can substantially reduce the effort required to generate all possible combinations of target keywords. For example, the expression before\s+(\w+\s+) + after[a-z]* matches any text that contains the term “before” followed by one or more space characters and one or more words before ending with a partial word “after” with trailing alphabets. This expression can detect various text such as ‘before and after’, ‘before and afterwards’ and ‘before this morning but after’. It is worth noting as we screened more articles, we systematically and progressively revised the existing terms and their weightings, taking into account additional information gathered.

### Validation of term-score screening using ASReview

We used ASReview [11] (version 1.0) to validate our screening approach described above. Typically, ASReview is calibrated from a small subset of pre-screened articles before actively learning the eligibility of articles while reviewers screen through the articles. In our case, we have screened all the articles using their derived term scores. Therefore, we let ASReview *independently* learn approximately 70% of the articles we screened before asking ASReview to rank the remaining 30% of the articles. We expected the ranking of articles by ASReview would reflect our screening decisions on those articles.

To prepare the input articles for ASReview, we split the screened articles into training and validation sets. Both sets contained information such as an article identifier, DOI, title, abstract and our screening decisions. The training set contains 63,752 articles (5,332 were marked as eligible; 58,420 were marked as ineligible). The validation set contains 27,119 articles. We removed all human decisions from the validation set before loading them into ASReview to sort and rank them based on the ‘learned’ relevance from the training set. To prevent the order effect during the training in ASReview, the training and validation sets were combined and shuffled so that the training and validation data were interspersed. In the input data file containing the training and validation sets, a row with a human decision represents a pre-screened article (training data), and a row without a human decision represents an unscreened article (validation data). The learning parameters used in ASReview were:

• Feature extraction technique: Term Frequency-Inverse Document Frequency (TF-IDF) (*Default*).

• Classifier: Logistic regression.

• Query strategy: Maximum (*Default*).

• Balance strategy: Simple sampling strategy.

Since ASReview was used for validation purposes in this study, we used the default values for most of the learning parameters, as these learning parameters (i.e., TF-IDF, Maximum query strategy) were computationally efficient, easy to interpret [26] and reproducible. The logistic regression classifier was chosen due to its binary nature in determining whether an article was eligible or not.

Although ASReview did not provide a score or prediction on whether an article is eligible or ineligible, it sorted the unscreened articles in the order of the ‘learned’ relevance from the training articles. We extracted the ASReview rankings of the 27,119 articles and compared the rankings with the term scores of the articles, as described in the Results section.

## Appendix 2 stopping criteria analysis using ASReview

To compare between our hybrid approach and screening with ASReview alone, we utilised ASReview’s inbuilt “simulate” functionality to mimic human screening with ASReview, using active learning with a small subset of pre-screened articles. The training and validation datasets used in the preliminary screening, along with all screening decisions, were provided to ASReview. We started the simulation with 20 pre-screened articles (as prior knowledge): 10 eligible and 10 ineligible articles. In each iteration, the simulation rank the unscreened articles based on these 20 articles, pick the top-ranked unscreened article and record the screening decision of that article (mimicking a human decision), re-rank the rest of the unscreened articles based on the decision and repeat. To ensure the ease of comparison, all seed articles were selected in the same order (not randomised) in all configuration profiles. Throughout this process, the simulation recorded the recall statistics (the top-ranked article in each iteration is an eligible article according to the human decision). The simulation halted when all the eligible articles were found. The simulation was run with default parameters except for the following:

• Prior included: 10.

• Prior excluded: 10.

• Classifier: Logistic regression (same value as the validation test above).

• Balance strategy: Simple sampling strategy (same value as the validation test above).

## Appendix 3 manual inspection of results

To cross-validate these configurations in Part 2, we manually inspected the top 500 and bottom 500 articles of these configurations. Supplementary Table 1 shows the accuracy and recall of each configuration (via F-score) for the eligible and ineligible articles among the top 500 and bottom 500 under each configuration. Term Score Ranking and Configuration D were able to accurately achieve high precision and recall (F-score = 1.0) in the bottom 500 articles compared to other configurations. The wide spread of eligible articles in Configuration A (see Fig. 3) resulted in its lower F-score compared to others. For the top 500, Configuration D identified the most eligible articles, followed by Configuration C and the term-scoring approach. Counterintuitively, Configuration C, which loaded 50 relevant and 50 irrelevant articles as seed articles, was able to identify more eligible articles compared to our term-scoring approach in the top 500 articles.

To investigate the ranking behaviour further across configurations, Supplementary Fig. 1 shows the ranking changes of 200 randomly selected articles from the 27,119 articles above. The rankings were re-scaled to between 0 and 10 to allow a meaningful comparison. On average, the ranking of an eligible article lowered (higher relevance), moving from Configuration A towards Configuration D. This suggests that the number of relevant and irrelevant articles included in the seed impacts the rankings. Specifically, the more relevant and informative the seeds, the better the rankings.

## Appendix 4 ASReview recall and stopping criteria analysis

Supplementary Fig. 2A shows a simulation of ASReview’s recall rate: the number of relevant records found based on the number of records screened. The number of relevant records (ground truth) was based on the decision from our term-score screening. ASReview found 95% (*n* = 8052) of the relevant articles after screening over 30,000 articles. The remaining 5% (*n* = 423) relevant articles were not found until after screening 84,475 out of 90,871 articles. If we could accept the loss of the 423 relevant articles, ASReview could potentially reduce the screening effort by 63%.

To demonstrate the problem of using the number of consecutive irrelevant articles as stopping criteria, Supplementary Fig. 2B shows the number of consecutive irrelevant articles screened before a relevant article was discovered in the same simulation above. While the number of consecutive irrelevant articles was considerably low at the beginning (i.e., a higher discovery rate of eligible articles), it increased exponentially towards the end. If we were to stop screening after seeing around 10 consecutive irrelevant articles (roughly screening 4000 relevant articles), we would have missed approximately 50% of the eligible articles; or 20% if we stopped after 40 consecutive irrelevant articles.

The results highlighted the strengths and nuances of existing semi-automated tools. In hindsight, if we had used the semi-automated tool, we could have saved considerable effort, as shown in the simulation. However, this retrospection is only possible because we preliminarily screened all articles using our term-scoring approach. In reality, if we rely on the semi-automated tool alone, it is challenging to determine when we have retrieved 95% of the eligible articles. Furthermore, the simulation also highlighted the nuance of the stopping criterion. This study demonstrated that prematurely stopping while using the tool can lead to a substantial loss of relevant articles in a review covering broad subject areas.
