# Supplementary material for: A hybrid approach to large-scale systematic literature reviews: combining automated tools with text-mining techniques
Source: BMC Res Notes. 2026 Jan 30;19:89. doi: 10.1186/s13104-026-07651-7 (PMC12930779; doi:10.1186/s13104-026-07651-7)
Supplement: Supplementary file 1 — Supplementary Material 1. [file 13104_2026_7651_MOESM1_ESM.docx]

**Supplementary Table 1** *The F-Score of Eligible and Ineligible Articles in the Top 500 and Bottom 500 of each Configuration*

|  | Top 500 F-Score (number of relevant articles identified out of 500) | Bottom 500 F-Score (number of irrelevant articles identified out of 500) |
| --- | --- | --- |
| Configuration A | 0.45 (146) | 0.94 (441) |
| Configuration B | 0.58 (202) | 0.99 (492) |
| Configuration C | 0.71 (276) | 0.99 (495) |
| Configuration D | 0.75 (297) | 1.00 (500) |
| Term Score Ranking | 0.69 (262) | 1.00 (500) |

*Note*. Configuration A used active learning with 1 relevant and 1 irrelevant article; Configuration B used rank-once mode with 20 relevant and 20 irrelevant articles; Configuration C used rank-once mode with 50 relevant and 50 irrelevant articles; Configuration D used rank-once mode with 5,332 relevant and 58,480 irrelevant articles.

**Supplementary Figure 1** *The Changes of 200 Randomly Selected Articles’ Rankings Across Different Configurations*


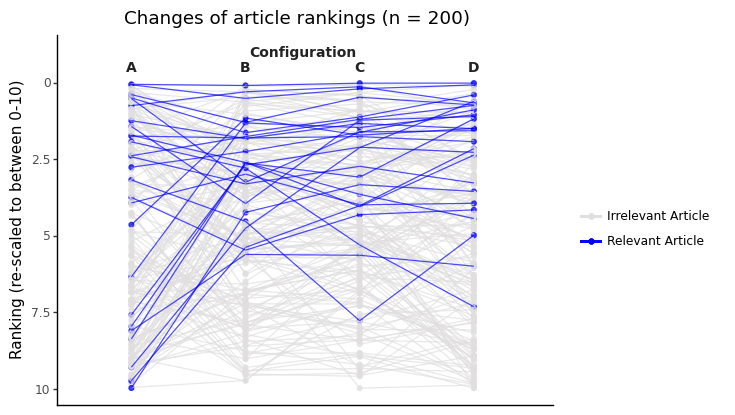

*Note*. The larger starting seeds can result in a higher ranking for the relevant articles. In Configuration D, we used the term-scoring approach to determine sizeable relevant/irrelevant articles as the seed articles.

**Supplementary Figure 2**: The Recall Rate (A) and Stopping Criterion (B) in a Simulation Using ASReview Based on the Manually Screened Articles


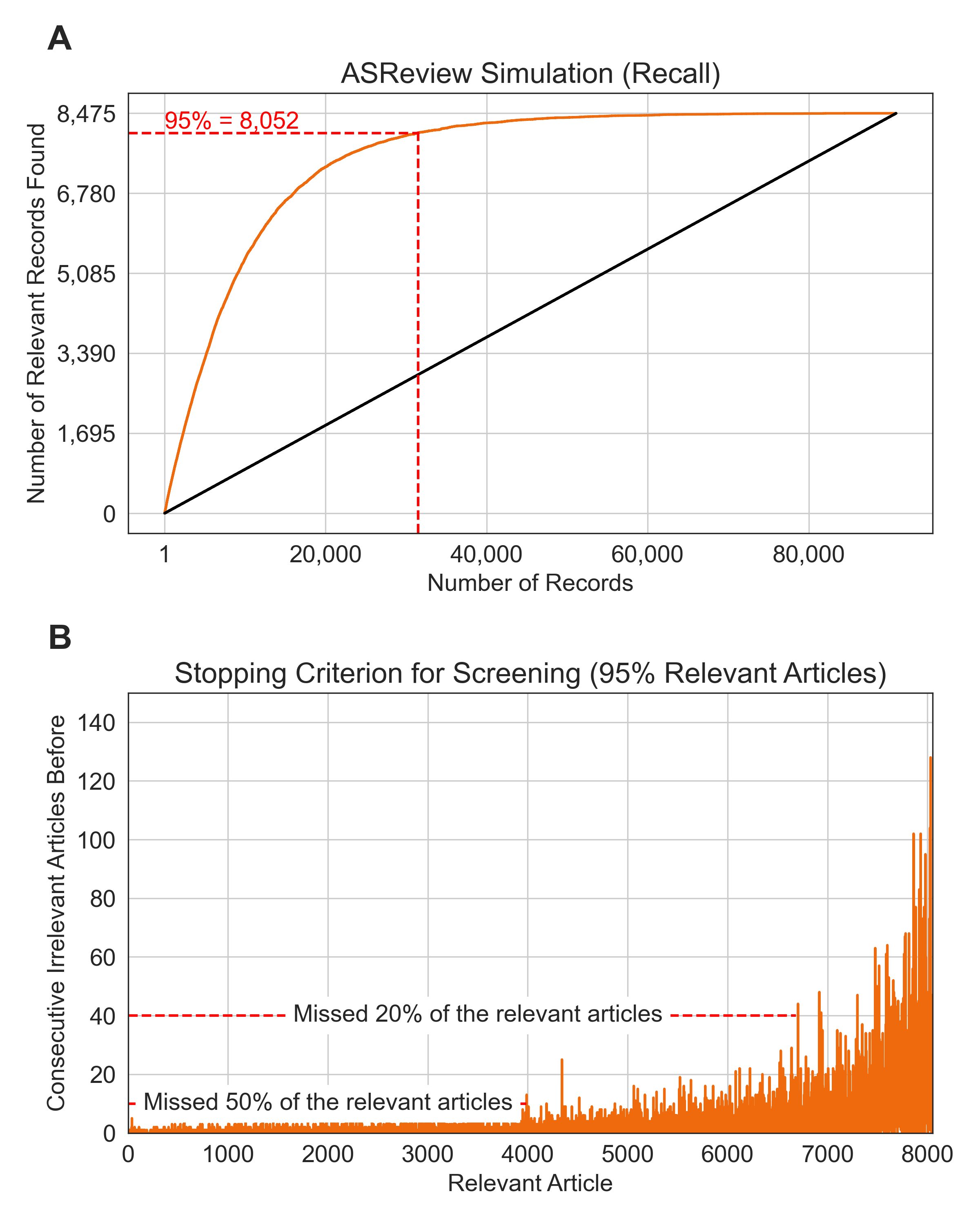

*Note.* ***A****: The orange line shows the number of relevant records found (y-axis) by ASReview after scanning a specific number of records (x-axis). The black line indicates the default performance of ASReview if no active learning/record prioritisation is involved. The result shows that ASReview (using the aforementioned learning parameters) found 95% of the relevant articles after screening over 30,000 articles, but the remaining 5% were not found until after screening 84,475 articles.* ***B****: If we were to stop screening after seeing around 10 consecutive irrelevant articles (approximately 4,000 relevant articles), we would have missed 50% of the eligible articles; or 20% if we stopped after 40 consecutive irrelevant articles.*
